# Supplementary material for: Are carbon emissions trading and green financial instruments synergistic? -Comprehensive quantitative research based on content analysis
Source: PLoS One. 2024 Mar 7;19(3):e0298601. doi: 10.1371/journal.pone.0298601 (PMC10919601; doi:10.1371/journal.pone.0298601)
Supplement: S1 Data — (ZIP) [file pone.0298601.s001.zip › Data/地级市Z_F工作报告.zip!/║╙▒▒/║Γ╦«╩╨/└┤╫╘╣1⁄2╓┌║┼í╛┬φ┐╦╩2╛▌═°í┐_2010.docx]

各位代表：

现在，我代表县人民政府向大会作工作报告，请予审议。并请县政协各位委员和列席会议的同志提出意见。

一、真抓实干，拼搏进取，超常发展迈出坚实步伐

刚刚过去的一年，是全县负重爬坡、克难奋进的一年。一年来，面对严峻的困难和挑战，我们在市委、市政府和县委的正确领导下，在县人大、县政协的监督支持下，团结带领全县人民，深入落实科学发展观，紧紧围绕“三创一超”目标，集智聚力，埋头苦干，圆满完成了县十四届人大四次会议确定的各项目标任务。

综合实力明显增强。全年完成生产总值45亿元，同比增长11%；完成全社会固定资产投资27.4亿元，增长48%，其中城镇固定资产投资16.4亿元，增长57 %；完成财政收入2.27亿元，增长28.1%，其中一般预算收入1亿元，增长55.2%；城镇居民人均可支配收入达到11735元，农民人均纯收入3980元，分别增长13.1%和7.2%。全县经济呈现出迎压上行、持续向好的良好态势。

工业经济提速发展。项目建设迈出新步伐。坚持以项目建设统揽工作全局，全年谋划建设1000万元以上项目64个，其中亿元以上10个，5亿元以上5个，省市重点项目13个。已开工项目53个，完成投资25.7亿元。投资10亿元的恒润复合管道项目顺利开工，标志着我县重大项目建设取得了突破。主导产业实现新提升。坚持把皮毛业繁荣振兴作为刻不容缓的首要任务，出台了一系列扶持政策，与桐乡、海宁对接，畅通内销渠道，成功举办了第十八届国际皮草交易会和首届大营皮草石家庄展销会。红运皮草、交易中心二期工程建成投入使用，启动了大营裘皮商城、物流中心等重点工程，产业发展稳步回升，一般纳税人企业由年初的160家，增加到247 家；实现外贸出口9822万美元，增长59倍；纳税达到2800万元，增长65%。玻璃钢和机械制造业在金融危机的压力下，转危为机，逆势走强，全年上交税金6000万元。园区建设取得新进展。玻璃钢园区北扩顺利实施，扩区方案通过省政府审批，规划面积由3平方公里扩大到9平方公里。启动了11平方公里的营新产业聚集区，道路框架已经完成。张新、马屯工业区建设加快，一批项目落地开工。

“三年大变样”成效显著。坚持抓关键、抓根本，完成了县城总体规划，规划面积由12平方公里扩大到27平方公里。县城水系、出口、人工湖、广场等专项规划已经启动。全年完成拆迁68万平方米，组织实施了总投资40亿元的39项重点工程。富强路、东环路、裕华街完成拓宽改造，启动了平原街、工业街西延工程和森林公园南湖建设。信誉楼商厦建成营业，东方夏威夷、帝景城等项目开工奠基。新建12.5公里的环城绿化带，城区新增绿地100万平方米，绿化覆盖率达到31%。廉租房和经济适用房完成主体工程，可解决522户群众的住房困难。县城污水处理厂建成运营，垃圾处理场正在加紧施工，今年可投入使用。城市功能和城市形象都跃上了新台阶。

农业农村工作扎实推进。国家强农惠农政策得到不折不扣落实，发放各类补贴6832万元。全年铺设防渗管道23万米，建成咸淡混浇井组140组，新增节水灌溉面积6.8万亩，粮食总产达到33万吨。全年植树75万株，造林8300亩，果品产量达到6.8万吨。新建规模奶牛养殖场3个，新增奶牛存栏2100头。斯格猪出栏21万头，特种养殖出栏52万只。城北110千伏、石村35千伏变电站建成运营。启动了联村集中供水工程，受益群众6.3万人。积极做好大广高速建设的协调工作，确保了工程顺利开展。完成了皮草环线二期、魏广路、秀贞路等140公里的乡村公路建设，城乡公路通车里程达到1100公里。

社会事业全面进步。认真落实促进再就业的各项扶助政策，新增就业岗位2000个。引导农村富余劳动力向非农产业转移，输出劳务9000人次。国家新型农村养老保险试点县获得批准，使全县农民终身受益。对城乡低保实行动态管理，全年发放低保金1000万元，发放五保供养金241万元。县城中心敬老院和马屯区域敬老院完成主体建设，集中供养人数可达860人。科技工作成绩突出，争取无偿资金376万元，开发省以上新产品6项。教育事业优先发展，高考成绩再创新高。文化事业提速推进，被评为“河北省文化先进县”。新型农村合作医疗健康运行，报免费用2900万元。完成大营、城关、恩察、唐林四个卫生院扩建工程，医疗条件得到改善。积极做好甲型流感防控，免费接种疫苗36300人次，有效控制了疫情蔓延。节能减排扎实推进，单位GDP能耗下降11%、COD排量削减率20.8%、二氧化硫排量削减率6.8%。高度重视安全生产，深入开展专项整治，保障了人民群众生命财产安全。加强社会维稳，全面落实县级干部包案责任制，建成城区视频监控系统，深入开展严打活动，圆满完成了国庆安保任务。同时，广播、档案、物价、审计、计生、人民武装等工作都取得了新成绩。

政府建设得到加强。扎实开展学习实践科学发展观、干部作风建设年和“骑单车、进农村”活动，深入基层，办实事，解难题。积极推进审批制度改革，行政审批项目由270项削减为142项，非行政审批项目由45项削减为23 项。自觉接受县人大的法律监督和县政协的民主监督，办理人大代表建议和政协委员提案187件，办复率100%。承诺的10件为民实事圆满完成，群众满意度不断攀升。

各位代表，过去的一年，是我县在严峻考验中突破力度最大的一年，科学发展转型最快的一年。全县上下付出了比以往更多的艰辛，破解了比以往更多的难题，在挑战中磨练了富民强县的坚强意志，在困难中积累了抢抓机遇的宝贵经验。成绩来之不易，这是县委正确领导和县四大班子齐心协力、合抓共推的结果，是各级干部服务大局、扎实工作的结果，是全县人民同心同德、团结奋斗的结果。在此，我代表县政府，向全县人民、向各位人大代表、政协委员和各位离退休老同志，向所有关心、支持、参与枣强发展的各界人士，致以崇高的敬意和衷心的感谢！

在肯定成绩的同时，我们也清醒地看到，我县经济社会发展中还存在一些矛盾和问题。一是工业投资不足，缺少立县的大项目、好项目。二是骨干企业创新能力不够，产品更新缓慢，制约了特色产业快速发展和转型升级。三是城市建设任务繁重，基础设施、特色塑造、城市管理与上级要求和全县人民的期盼还有较大差距。四是农业产业化水平低，农民增收难度加大。五是财政收入质量不高，收支矛盾十分突出。从政府自身看，执行力和落实力还有待进一步提高，工作作风还有待进一步转变，发展环境还有待进一步优化。对此，我们将高度重视，切实采取措施，认真加以解决。

二、突出重点，锐意进取，奋力开创2010年工作新局面

2010年是实现“十一五”目标的冲刺之年，也是实施“十二五”规划的奠基之年，更是实现枣强科学超常发展的关键之年，做好今年的工作意义十分重大。

当前，全国经济企稳向好，发展形势好于去年，国家继续实施积极的财政政策和适度宽松的货币政策，为我们加快发展创造了良好外部环境；我县被纳入“水市湖城”战略框架，实行同城化管理，为我们加速城镇建设带来了前所未有的机遇；经过去年城镇规划修编，发展空间进一步拉大，为项目落地和城镇扩张提供了有利条件；大广高速通车在即，邯黄铁路启动建设，使我县区位优势更加明显；特别是近几年开工建设的一批项目将在今年逐步投产达效，皮毛业走出了最困难的低谷，全县经济开始进入良性发展轨道，各级干部和企业家思想深度解放，争先创优的意识不断增强，干事创业的热情不断高涨。这些，都为我们加快发展奠定了坚实基础。同时，也要看到，金融危机的影响依然存在，加快发展仍存在诸多不确定、不稳定因素。挑战与机遇并存，危机与希望同在。我们必须进一步增强使命感和紧迫感，以更加开放的姿态、更加饱满的热情、更加有力的措施，奋发有为，求实创新，努力开创各项工作新局面。

根据县委九届五次全会精神，今年政府工作的总体思路是：以科学发展观为指导，以加快经济发展方式转变为主线，进一步唱响“三创一超”主旋律，更加注重优化产业结构和重点项目建设、更加注重提升城市功能和改善生态环境、更加注重增强经济实力和缩小城乡差别、更加注重改善民生和促进社会和谐，深入实施项目攻坚年、产业提升年、城镇建设年，推动经济社会科学超常发展。

全年主要预期目标是：生产总值增长12%以上；财政收入增长25%以上，力争突破3亿元，其中一般预算收入增长30%以上；全社会固定资产投资增长40%以上，其中城镇固定资产投资增长50%以上；城镇居民人均可支配收入增长12%，农民人均纯收入增长7.2%。

实现以上目标任务，必须重点做好以下六个方面的工作。

（一）扎实推进项目攻坚年，努力增强经济发展新动力

狠抓大项目建设不动摇。坚持把大抓项目、抓大项目作为经济工作的核心，以“三四”竞赛活动为目标，力争年内新开工亿元以上项目40个，其中10亿元以上4个；谋划推进亿元以上项目40个，其中10亿元以上4个；新签约亿元以上项目40个，其中10亿元以上4个。着力抓好“项目攻坚月”签约项目的跟进，中建材钢结构、爱特佳尼克服、铁路专用线缆、瑞星燃气输配基地、鑫山输送机械、晶美减速机等项目力争早日开工落地，年内完成主体工程；铁路机车配件、恒发复合材料、烟气脱硫除尘、交易中心三期等项目，尽快落实资金、地块，早日启动建设。加快推进中国多肽工业园项目，力争年内开工。对恒润复合管道一期工程、大营裘皮商城、物流中心等在建项目，倒排工期、挂图作战，力促年内竣工。结合“十二五”规划编制，深入研究国家产业政策，谋划一批立县的大项目、好项目，全年争列省重点项目10个以上。

锲而不舍外引战略投资者。坚持把引进战略投资者作为做大经济总量、调整经济结构的第一要务。进一步完善招商引资政策，强化目标管理，创新招商方式，大力推进“产业链”招商、“企业链”招商、小团组招商、亲情招商、以商招商，努力引进一批重大骨干项目，在大招商、招大商上实现新突破。进一步做好借力京津这篇大文章，研究京津、对接京津、服务京津，力争取得更大成效。

千方百计激活民间投资。在抓好招商引资的同时，更加积极主动地引导和鼓励民间投资。大力开展全民创业和二次创业活动。全民创业，在工商税收优惠、财政资金支持、行政性收费减免等方面，加大扶持力度，激发创业热情、放开创业领域、提供创业条件，努力调动民间资本投资的积极性。二次创业，促进民营企业家进一步解放思想，大力开展“1+1”活动，加强与大企业、大集团合作，主动融入高端产业链条，力争在短时间内实现民营企业的再造。

抢抓机遇建好工业园区。利用新一轮土地规划修编，按照“产业特色化、布局集群化、环境生态化”的要求，着力打造第三代产业园区。玻璃钢园区北扩作为今年的首要任务、重点工程，要尽快制定园区详规，启动2平方公里的起步区建设，加快富强路、胜利路北延和振华街建设，构建以建设路为中心的“四纵两横”路网框架，为项目入驻创造条件，年内进区亿元以上项目6个。营新产业聚集区加快启动精品裘服加工园，完成路网建设和分区规划，入园项目8个以上。张新、马屯工业区加大招商引资力度，尽快形成规模优势。对入区项目，严把投资强度关，提高容积率、产出率，坚决杜绝低水平重复建设。

全力以赴加快项目推进。完善重点项目考核奖惩机制，明确目标、卡死责任、严格落实。继续实行重点项目县级领导分包责任制，为项目建设搞好全程服务。多渠道破解土地瓶颈。做好大项目、好项目的谋划申报，最大限度争取土地指标；整理、复垦废弃地，加大置换力度；对批而未建、占而不建的土地，坚决依法收回；加快新民居建设，腾出更多项目用地。

（二）扎实推进产业提升年，努力增强主导产业发展新优势

以更新的理念推进皮毛业转型升级。围绕构建“中国裘皮名城”，以城市建设为载体，以市场开发为依托，以骨干企业为龙头，以发展服装加工为重心，努力打造全国最大的裘皮产业集群。完善规划促提升，尽快完成发展规划，力争3年实现产值超百亿、纳税超亿元，5年建成完备的产业链条和市场体系，实现年产值200亿元以上，成为名副其实的“中国裘皮名城”。坚持大营、新屯一体发展、同步推进，扩大战略空间。对大营城区进行深度规划，年内完成城区总规，做好市场、道路、重要节点的控制性详规，启动 一批市场、物流、商住、路街建设工程，力求单体出精品，整体出形象。拓展市场促提升，加快大营裘皮商城建设，启动交易中心三期工程，谋划建设生皮、辅料市场。办好石家庄、太原、济南等地的展销活动，积极开拓国内市场。推进与俄罗斯马加丹州合作项目尽快投产达效，加强与波兰马克西姆公司合作，巩固拓宽国际市场，努力形成“内销外贸并举，多元市场支撑”的良性格局。做强骨干促提升。筛选10家规模企业，跟踪帮扶，重点支持，促进企业做大做强，力争纳税超200万元企业达到5家以上。培育品牌促提升。加快省级皮毛检测中心和裘皮服装出口检疫示范区建设，强制企业按标准生产。鼓励现有企业与行业龙头合作，贴牌生产，借势发展。大力引进国内外知名企业投资建厂，引进先进的工艺设备和经营理念，提高研发、设计水平。积极发展自主品牌，“南极公主”、“弗兰尼”争创省著名商标。创优环境促提升。强化扶持皮毛业发展的各项措施，鼓励企业解放思想，放手放胆发展。创新体制，完善职能，放宽政策，放大权限，促进皮毛业健康、跨越发展。力争全年实现产值40亿元，纳税突破6000万元。

以更强的举措推进玻璃钢、机械制造业做大做强。优化产品结构。组建国家级玻璃钢产品检测中心，扎实开展与同行业先进水平的“对标”行动，促进产品向标准化、定型化发展。鼓励企业瞄准市场前沿，积极开发航空航天、交通运输、家居民用等发展潜力大的新产品。着力实施“一企一品”战略，引导企业向专业化、规模化迈进。整合资源，努力打造全国最大的玻璃钢管道、模压产品生产基地。培育龙头企业。支持重点企业提升单体规模，增强核心竞争力，力争纳税300万元以上企业达到15家、2家突破千万元、5家进入全市重点企业支持目录。鼓励企业上市，恒润集团力争年内完成前期申报工作。争创知名品牌。“恒润”、“冀峰”、“巨鑫”争创中国驰名商标，“恒圣合”、“黑龙港”争创省著名商标。精心组织各类展示展销活动，不断提高知名度和影响力。力争玻璃钢业全年实现产值50亿元，机械制造业实现产值25亿元，两大产业全年纳税超过8000万元。同时，围绕国家产业政策，积极发展高新技术、文化创意、绿色健康、生态观赏等现代产业，培育新的经济增长极。

以更大的决心推进可持续发展。皮毛业，加大对鞣制、硝染企业的治理力度，引导企业向园区集中，实现集中治污、达标排放。玻璃钢业，积极探索下脚料回收利用办法，最大限度减少废料产生。加快县城生活垃圾处理场建设，确保年内启用。完善垃圾处理费和污水处理费征收机制，促进两场（厂）高效运转。全年单位GDP能耗下降11%，COD排量削减148吨，二氧化硫排量削减32吨，圆满完成“十一五”目标任务。

同时，高度重视工业运行调节，加大对重点行业、重点企业的监测，搞好煤、电、油、运供应，及时解决工业运行中的各类问题。

（三）扎实推进城镇建设年，努力塑造城镇面貌新形象

加速推进县城建设。以构建独具特色的“滨湖生态园林城市”为目标，按照“拆建并重、以建为主、建管结合”的要求，着眼“改造旧城、拓展新城、打造景区”，统筹实施“5路2桥”和“3132”工程，分旧城（以枣强新河为中心）和新城（以索泸河、森林公园为中心）两个板块，同步推进。旧城，坚持拆迁、建设、管理并举。拆迁，按照“四个转变”的要求，研究制订城中村、旧居住区拆迁改造实施办法，强化措施，积极推进，确保完成拆迁50万平方米。建设，以枣北拆迁区域为重点，加快帝景城、东方夏威夷两个住宅小区建设，启动城北广场、五星级酒店项目，努力建成城市的标志性区域。抓好新华街、胜利路便道硬化、绿化，积极推进集中供热和天然气进城工程，合理规划公厕和垃圾箱布局，继续实施小街小巷改造，进一步完善城市功能。管理，切实维护规划刚性，严格审批程序，坚决打击非法建设。加强对项目招标、工程质量的监管，严厉打击虚假招标、围标串标、倒手转包、违法分包行为。实施城市容貌整治攻坚行动，对市容卫生、夜景照明、交通秩序、建筑立面、广告牌匾、马路市场进行专项整治，打造更加整洁、优美的城市环境。新城，以规划为先导，以水网、路网建设为重点，加快开发建设。规划，完成新城重点片区的修建性详规和水网、路网、绿网、管网等专项规划，确保高标准起步。水网，启动索泸河县城段改造工程，加快南湖、西湖建设，构建新城水系，着力打造独具魅力的城市景区。路网，借助肃临线升级，启动迎宾路（西外环）建设，年内完成前期准备工作。完成中华街（工业街）、平原街西延，建设两座新桥，打通断头路，拉开新城框架。启动城市规划展览馆建设，力争年内完成主体工程。规划启动大型物流市场，提升城市功能。逐步引导行政办公、生态人居、商贸物流、卫生教育等要素向新城聚集，努力建设宜居、宜业、宜商、宜学、宜游的生态新城。深入挖掘“董子故里”、“革命老区”、“道德高地”文化底蕴，加大宣传推介力度，把“滨湖生态园林城市”叫响做名。

全力提高城镇聚集能力。把转移农民进城作为推进城市化的一项战略重点，大力发展城市产业，为农民进城提供就业机会，降低农民进城创业成本，全年新增城镇人口1万人。加强融资平台建设，提高融资水平，为城市建设提供资金保障。坚持政府主导、市场主体，完善土地储备供应机制，科学有序供地，实现城镇建设投资与收益的良性循环。

同时，按照“1+2”空间发展战略的要求，抓好大营、肖张的规划建设。肖张镇要按照高新园区、休闲采摘、商贸物流三个功能分区，启动总体规划，努力建设“水市湖城”的观光基地和物流中心。

（四）扎实推进现代农业建设，努力拓展农民增收新空间

积极调整农业结构。稳定粮食生产，突出抓好60万亩科技示范田建设，确保粮食总产达到34万吨。着力实施“百千兴业工程”，新建标准化奶牛小区3个，优质奶牛存栏达到6000头。斯格猪出栏13万头，特种养殖出栏60万只。努力促进林业由用材林向经济林转变，全年植树80万株，造林9500亩，发展优质果品3000亩，果品产量达到 7万 吨。

大力推进农业产业化。以工业化的思维指导农业发展，集中培育果品、粮油、蔬菜加工业，力争新上投资5000万元以上农业产业化项目3个。积极推广大王村农业服务中心、芍药农业合作社成功经验，加快建设一批生产型、服务型、销售型的专业合作组织，年内达到30家。抓好农业品牌建设，阳谷庄甘薯、芍药蔬菜尽快拿到无公害基地环评认证，全县农产品注册品牌达到10个以上。

切实加强农业基础设施建设。借助大广高速、邯黄铁路用土机遇，扩挖坑塘，增强蓄水能力。加快实施河渠改造工程，新增“引黄农灌”面积6万亩。全年发展咸淡混浇井组200组，铺设防渗管道13万米。继续推进联村集中供水，完成大营、新屯水厂建设，受益群众10万人。加快肃临路、武馆路升级的前期准备，争取早日开工。实施枣吉路改造，加快肖马路、魏广路二期和村级路建设，完成肖马路屈纸房桥、西臣赞桥的新改建，新增通车里程70公里。

着力推进新民居建设。按照农业集约化、农村社区化、生活城市化的要求，以临近产业聚集区、主干公路和乡镇政府所在地为重点，采取联村并居、中心村聚集、村企共建等模式，力争三年完成75个新民居示范村建设。今年重点推进42个村的规划建设，每个乡镇至少抓好1个联村并居试点，确保年内出形象、见成效。

（五）扎实推进服务业发展，努力促进第三产业迈出新步伐

结合三年大变样，加强粮食、家具、建材等专业市场建设，逐步构建布局合理、功能完善、设施一流的现代市场体系。加大服务业招商引资力度，吸引国内外知名零售企业入驻枣强。着力抓好大营物流中心、巨石物流中心等项目建设，打造物流龙头企业。深入实施“万村千乡”、“农超对接”市场工程，全面落实家电、汽车、摩托车“三下乡”和“以旧换新”政策，进一步开拓消费市场。大力发展文化娱乐、体育健身、养老托老、家政服务等产业，促进服务消费。加强银企合作，扩大金融产品应用，重视担保机构建设，积极推行联保贷款，增强融资能力。加快旅游基础设施建设，主动融入衡水湖生态旅游圈。全力办好大营皮草交易会，打造会展品牌。

（六）扎实推进各项民生工程，努力开创更加和谐稳定新局面

努力扩大就业。认真落实促进就业的各项政策措施，多渠道增加就业岗位。深入实施城乡技能就业扶助计划，完成就业培训5000人次、农村实用技术培训1万人次。确保新增城镇就业2000人，输出劳务1万人次，登记失业率控制在4%以内。

完善社会保障。推进社会保险扩面征缴，将更多灵活就业人员纳入社保范围。完善企业养老保险制度，稳妥推进县社、卫生系统的企业改革。积极做好新型农村养老保险试点工作，参保率达到90%以上。落实好城乡居民最低生活保障，实现应保尽保、按标施保。完成县城中心敬老院和马屯区域敬老院配套建设，五保供养率达到50％以上。

强化科技支撑。加大科技推广力度，争创全国科普示范县。鼓励企业加强与大专院校、科研单位的密切合作，支持企业建立技术研发中心，全年开发省以上新产品7项，争取上级支持资金600万元。

优先发展教育。巩固学前三年教育成果，把一幼建成省级示范性幼儿园。提高教学质量，加大高中教育普及力度，入学率达到80%以上。加快职教中心建设，提高办学水平。

重视文化事业。加强文物保护管理，力争完成董仲舒石像保护室建设。支持做好《天下裘都》电视剧拍摄，打造文化品牌。深入实施有线电视“村村通”，年内发展100个村、1.5万户。

改善医疗卫生。启动县医院改建，努力建成全市一流的区域中心医院。加快卫生信息化建设，逐步建立居民健康档案。深化新型农村合作医疗，抓好门诊统筹，提高补助标准，参合率达到90%以上。

做好人口计生工作。落实人口和计划生育政策，实施“计生惠民”行动和优生促进工程，努力提高人口素质。继续实行计划生育目标管理责任制，稳定低生育水平。扎实做好第六次人口普查工作。

维护安全稳定。加大安全生产监察执法和监管力度，强化烟花爆竹、危险化学品、人员密集场所、道路交通等专项整治，确保不发生重大安全事故。高度重视信访工作，健全矛盾排查调处机制。加强社会治安综合治理，严厉打击各类刑事犯罪。完善公共应急管理，提高处理突发事件能力，切实维护社会和谐稳定。

同时，认真做好国防动员、审计、统计、物价、档案等工作。

三、恪尽职守，执政为民，努力打造人民满意政府

解放思想，提高政府创新力。创新理念，善于用超前的思维研究发展思路，完善发展措施，使各项工作更具前瞻性和开拓力。创强举措，打破陈规陋习，用改革的办法、市场的手段，解决前进中的矛盾和问题。创优成果，坚持工作高标准、干事严要求，追求卓越、争先创优，好中求好、精益求精，奋力开创各项工作新局面。

真抓实干，提高政府执行力。对出台的政策、议定的事项说到做到，说好做好，实干实效，大力营造“说实话、办实事、求实效”的清新政风。坚持工作落实到项目上、项目落实到责任上、责任落实到考核上、考核落实到奖惩上。进一步细化分线作战、协同推进的工作体系，对重点工作、重大项目，逐个分解立项，明确责任要求，加强督查问责，确保各项工作扎实推进。

依法行政，提高政府公信力。自觉接受人大、政协及社会各界的监督，坚持用制度管权、管人、管事。完善重大事项集体讨论、专家咨询、听证公示制度，提高决策的科学化、民主化水平。严格落实行政执法责任制，加大对不作为、慢作为、乱作为的整治力度，克服“中梗阻”、打破“潜规则”，切实做到办事高效、运转协调、政令畅通。

廉洁勤政，提高政府凝聚力。深入落实党风廉政建设责任制，自觉遵守廉洁自律的各项规定，勤勉尽责、秉公用权，堂堂正正做人，清清白白为官，干干净净做事。珍惜民资民力，勤俭办一切事情，挤出更多财力为群众谋利，为百姓造福，办好为民实事，以实际行动和工作业绩，努力建设团结务实、廉洁高效的人民政府。

各位代表，新的一年，困难和挑战考验着我们，责任和使命激励着我们。让我们在中共枣强县委的坚强领导下，在县人大、县政协的监督支持下，鼓足实干之劲，凝聚和谐之力，团结一心，突破奋进，为创造枣强更加美好的明天而努力奋斗！
